# Association between fertility and HIV status: what implications for HIV estimates?

**DOI:** 10.1186/1471-2458-8-309

**Published:** 2008-09-11

**Authors:** Eugene J Kongnyuy, Charles S Wiysonge

**Affiliations:** 1 Child and Reproductive Health Group, Liverpool School of Tropical Medicine, Liverpool, UK; 2 South African Cochrane Centre, South African Medical Research Council, Cape Town, Republic of South Africa

## Abstract

**Background:**

Most estimates of HIV prevalence have been based on sentinel surveillance of pregnant women which may either under-estimate or over-estimate the actual prevalence in adult female population. One situation which can lead to either an underestimate or an overestimate of the actual HIV prevalence is where there is a significant difference in fertility rates between HIV-positive and HIV-negative women. Our aim was to compare the fertility rates of HIV-infected and HIV-uninfected women in Cameroon in order to make recommendations on the appropriate adjustments when using antenatal sentinel data to estimate HIV prevalence

**Methods:**

Cross-sectional, population-based study using data from 4493 sexually active women aged15 to 49 years who participated in the 2004 Cameroon Demographic and Health Survey.

**Results:**

In the rural area, the age-specific fertility rates in both HIV positive and HIV negative women increased from 15–19 years age bracket to a maximum at 20–24 years and then decreased monotonically till 35–49 years. Similar trends were observed in the urban area. The overall fertility rate for HIV positive women was 118.7 births per 1000 woman-years (95% Confidence Interval [CI] 98.4 to 142.0) compared to 171.3 births per 1000 woman-years (95% CI 164.5 to 178.2) for HIV negative women. The ratio of the fertility rate in HIV positive women to the fertility rate of HIV negative women (called the relative inclusion ratio) was 0.69 (95% CI 0.62 to 0.75).

**Conclusion:**

Fertility rates are lower in HIV-positive than HIV-negative women in Cameroon. The findings of this study support the use of summary RIR for the adjustment of HIV prevalence (among adult female population) obtained from sentinel surveillance in antenatal clinics.

## Background

The prevalence of Human Immunodeficiency Virus (HIV) is a measure of disease burden which is important in evaluating HIV prevention programmes and in designing new preventive strategies. Most estimates of HIV prevalence in developing countries have been based on sentinel surveillance of pregnant women [1]. HIV prevalence among pregnant women in antenatal clinics (ANC) closely approximates HIV prevalence in the adult population. ANC data is sometimes used to estimates HIV prevalence in general female population, instead of the general adult population (both males and females). This assumption is, however, unfounded [2]. Women who adopt abstinence or correct and consistent condom use in response to HIV prevention programmes are less likely to be pregnant and equally less likely to contract HIV infection. As such, sentinel surveillance of pregnant women would over-estimate HIV prevalence in the general female population. On the other hand, there is some evidence that HIV-1 could impair fertility [3,4]. If HIV infected women are less likely to be pregnant because of impairment of their fertility by the HIV virus, the use of sentinel surveillance would under-estimate the HIV prevalence.

Research comparing ANC estimates of HIV prevalence with HIV prevalence from population-based studies has shown that ANC figures closely estimate the actual population prevalence [5-7]. However, the findings have not been consistent [8]. Saphonn et al (2002) reported that although the prevalence of HIV in ANC was good for monitoring trends, it over-estimated HIV prevalence in rural Cambodia [9]. On the contrary, Changalucha et al (2002) reported that the prevalence of HIV from ANC underestimated community HIV prevalence in Tanzania [10]. Grassly, et al (2006) reviewed studies that have been carried out in different countries and compared estimates from ANC sentinel surveillance with population based estimates [11]. They found that in 12 out of 15 countries, HIV prevalence from ANC surveillance closely estimated the actual population prevalence. However, prevalence from ANC over-estimated community prevalence in Kenya and Rwanda, but underestimated the community prevalence in Zambia. Assuming that prevalence from ANC surveillance is representative of pregnant women, and that errors resulting from laboratory testing are minimal, one major explanation of inaccuracies in the estimates is the differences in the fertility rates between HIV positive and negative women. The accuracy of HIV estimates from ANC also depends on the contraceptive prevalence and ANC coverage.

Improved methods for estimating HIV prevalence such as population-based surveys can provide useful information on HIV prevalence levels and distribution, and improve HIV estimates at national and regional levels [12]. However, surveys may be costly especially when there are frequent and nation-wide.

Research on ANC sentinel surveillance has mainly been focused on the role of behavioural data in ANC surveillance [13], uses of prevalence data [14], coverage [15], assessing the trends of HIV prevalence [16-18], comparison between HIV prevalence from ANC and community prevalence [9,10] and methods of adjustment of estimates from ANC surveillance [11,19]. In addition some research has addressed the differences in fertility according to HIV status [2,20]. Glynn et al (2000) reported that the birth interval was reduced in HIV positive multiparous women compared to HIV-negative multiparous women in three African cities, namely Yaounde (Cameroon), Kisumu (Kenya) and Ndola (Zambia) [20].

Using sentinel data of pregnant women, the prevalence of HIV in Cameroon rose from 0.5% in 1987 to 10.8% in 2000 and then dropped to 7.3% in 2002 (Ministry of Public Health, 2001 and 2003) [21]. A population-based survey conducted in 2004 estimated the overall adult HIV prevalence in Cameroon to be 5.5% [22]. It is not clear whether this represents a true decrease in the prevalence of HIV or an artifact due to the differences in the two methods of data collection. Knowledge about the accuracy of sentinel prevalence as a proxy for national HIV prevalence is indispensable especially in a country facing rapid changes in the HIV prevalence. Differential prevalence of HIV in pregnant and non-pregnant women has been reported to be significantly associated with age, marital status, parity, schooling, and contraceptive use [23].

The purpose of our study was to compare the fertility rates of HIV-infected and HIV-uninfected women using population-based data in order to make recommendations on the appropriate adjustments when using sentinel data in designing and evaluating HIV prevention programmes in Cameroon.

## Methods

### Design

This study is a cross-sectional, population-based survey that uses data from the 2004 Cameroon Demographic and Health Survey (DHS) [22]. Women of reproductive age (15 to 49 years) were interviewed about their past and current reproductive history and tested for HIV after obtaining informed consent. Ethical approval was sought from the Cameroon Ministry of Health prior to the original survey [22].

### Population and sampling

The survey used a two stage cluster sampling technique. The sample frame was a list of all Enumeration Areas (clusters) established by a General Census of Population and Housing in 2003. The first stage involved selecting 466 clusters (primary sampling units) with a probability proportional to the size, the size being the number of households in the cluster. The second stage involved the systematic sampling of households from the selected clusters. All women aged 15 to 49 years in the selected households were interviewed. The details of the study methods have been published elsewhere [22]. The current report included only sexually active women aged 15 to 49 years.

### Laboratory analysis

The HIV status was screened by direct ELISA test (Genscreen Plus Version, BioRad Laboratories) and confirmed by a competitive ELISA test (Wellcozyme HIV-1 recombinant, ABBOTT, specific for HIV 1) and a rapid test (Determine, ABBOTT, specific for HIV 2). Only positive samples (with direct ELISA) were confirmed with competitive ELISA and only discordant samples were tested with Determine. All positive results in this survey were HIV-1: no HIV-2 was detected. For internal quality control, 5% of the HIV negative samples on direct ELISA were confirmed by competitive ELISA and Determine. Details of the testing algorithm has been published elsewhere [22].

### Definitions

#### Fertility rates

We define the general fertility rate in this study as the total number of births in the 36 months preceding the survey divided by the total number of woman-years of exposure during that period (36 months) multiplied by 1000 [24]. The age-specific fertility rate is the value of fertility rate for seven five-year groups (15–19, 20–24, 25–29, 30–34, 35–39, 40–44 and 45–49 years). Woman-years of exposure is the sum of the number of months exposed in the five-year age bracket during the time period divided by 12.

#### Relative inclusion ratio

The relative inclusion ratio (RIR) is the ratio of the fertility rate in HIV positive women of reproductive age to the fertility rate of HIV negative women of reproductive age (15–49 years). Nicoll et al used this ratio to compare the relative fertility in HIV infected and uninfected women, so as to determine the relative likelihood of including these two groups of women in a seroprevalence survey in antenatal clinics [2]. A ratio of 1.00 suggests a good estimation; a ratio of less than 1.00 indicates an underestimation and a ratio of more than 1.00 is an over-estimation of the HIV prevalence in the general population. ANC-based HIV prevalence can be adjusted for the effect of differential fertility rates by using the formula "Adjusted HIV Prevalence = {Unadjusted HIV Prevalence}/{RIR}". For example if the Unadjusted ANC-HIV Prevalence is 7.5% and RIR is 0.75, then the Adjusted HIV Prevalence is 7.5/0.75 or 10.0%.

#### Wealth index

Wealth index was used as our measure of socio-economic status. A score was given to each available household amenity based on the Health, Nutrition and Population/Poverty Thematic Group of the World Bank [25]. The total score for each household constituted the wealth index score for that household. Each woman was assigned the wealth index score of her household. From this score we distinguished three equal-sized classes of participants according to their wealth, based on percentiles (<33.33 percentile, 33.33 to 66.66 percentile and >66.66 percentile).

### Statistical Analyses

All cases in the DHS data are given weights to adjust for differences in probability of selection and to adjust for the non-response in order to produce the proper representation. Individual weights were used for secondary data analysis in this study. Data were analyzed using SPSS version 13.0 for Windows. The results are reported as rates or ratios with their corresponding 95% confidence intervals (CI) and a p-value less than 0.05 was considered significant.

## Results

### Characteristics of the study population

Overall, 4493 sexually active women participated in the survey, giving a response rate of 92.1%. Of this number, 336 (7.5%) were HIV positive. Table 1 presents the characteristics of the study population by HIV status. The mean age of women and the mean age at first intercourse were not significantly different between HIV positive and HIV negative women. The median parity was 2 (range 0 to 12) for HIV positive and 2 (range 0 to 14) for HIV negative women (p < 0.001). The prevalence of HIV infection was significantly higher in wealthier and more educated women (p < 0.001 for each). The prevalence also varied according to the marital status: 3.5% amongst women who have never married, 6.2% amongst currently married and 18.5% in divorced or widowed women (p < 0.001).

**Table 1:** Distribution of socio-demographic characteristics of Cameroonian women of reproductive age by HIV status

***Characteristic***	***HIV positive N (%)***	***HIV negative N (%)***	***p-value***
Mean age, years (SD)	29.3 (7.6)	28.9 (9.1)	0.304
Median age at first intercourse (range)	16(10–26)	16 (8–33)	0.167
Median parity (range)	2 (0–12)	2 (0–14)	<0.001
Median number of living children (range)	2 (0–9)	2 (0–12)	<0.001

***Place of residence***			

Urban	228 (9.5)	2183 (90.5)	<0.001
Rural	108 (5.2)	1973 (94.8)	

***Marital status***			

Never married	37 (6.5)	529 (93.5)	<0.001
Currently married	218 (6.2)	3274 (93.8)	
Formerly married	80 (18.5)	353 (81.5)	

***Education***			

No school	37 (3.5)	1035 (96.5)	<0.001
Primary	143 (8.1)	1619 (91.9)	
Secondary/higher	155 (9.4)	1502 (90.6)	

***Religion***			

Christians	276 (8.7)	2896 (91.3)	<0.001
Muslims	45 (5.5)	778 (94.5)	
Others	15 (3.0)	483 (97.0)	

***Wealth index***			

low	57 (3.8)	1441 (96.2)	<0.001
middle	132 (8.8)	1368 (91.2)	
high	146 (9.8)	1350 (90.2)	

HIV = Human Immunodeficiency Virus

### Fertility rates

Table 2 presents the age-specific fertility rates for rural areas. In the rural area, the fertility rate in both HIV positive and HIV negative women increased from 15–19 years to a maximum at 20–24 years and then decreased monotonically till the 35–49 years. However, in all age brackets the fertility rate was lower in the HIV positive compared to the HIV negative women. The overall fertility rate was 157.7 births per 1000 woman-years (95% CI 118.4 to 204.1) in HIV-infected women compared to 243.7 births per 1000 woman-years (95% CI 229.7 to 258.2) in uninfected women. Consequently, all relative inclusion ratios (RIRs) were lower than unity, and the overall RIR was 0.65 (95% CI 0.59 to 0.71).

**Table 2:** Fertility rates and relative inclusion ratios (RIR) for HIV positive and HIV negative women by age in the rural area of Cameroon, 2004.

**HIV positive women**				**HIV negative women**			
Age bracket (years)	Number of births **(A1)**	Woman-years at risk **(B1)**	Fertility rate (95% CI) C1 = (A1/B1)*1000	Number of births **(A2)**	Woman-years at risk **(B2)**	Fertility rate (95% CI) C2 = (A2/B2)*1000	RIR (95% CI) **C1/C2**

15–19	4	43.4	92.2 (30.3–209.3)	238	1518.8	156.7 (137.7–177.6)	0.59 (0.51–0.67)
20–24	14	72.0	194.4 (110.7–318.5)	347	1316.7	263.5 (236.9–292.4)	0.74 (0.68–0.79)
25–29	14	89.3	156.8 (89.2–256.8)	234	987.0	238.1 (209.0–237.7)	0.66 (0.60–0.72)
30–34	9	61.0	147.5 (72.0–270.8)	167	815.3	204.8 (175.5–237.7)	0.72 (0.66–0.78)
≥ 35	3	56.7	52.9 (13.5–144.0)	141	1506.4	94.3 (79.7–110.75)	0. 56 (0.46–0.67)

**Total**	44	279.0	157.7 (118.4–204.1)	1127	4625.4	243.7 (229.7–258.2)	0.65 (0.59–0.71)

CI = confidence interval; RIR = relative inclusion ratio; HIV = Human Immunodeficiency Virus

Table 3 compares the age-specific fertility rates of HIV positive and HIV negative women in urban areas. Similar trends are observed as in rural areas: fertility is higher in HIV negative than in HIV positive women in all but for the 15–19 year age brackets. However, the peak of fertility in urban areas is reached at a later age (25–29 years) than in rural areas (20–24 years). Like in rural areas, the overall fertility is lower in HIV positive (102.7 births per 1000 woman-years, 95% CI 80.7 to 129.0) than in HIV negative women (124.0 births per 1000 woman-years, 95% CI 116.0 to 1312.4) and consequently the relative inclusion ratio is low (0.83, 95% CI 0.76 to 0.89).

**Table 3:** Fertility rates and relative inclusion ratios (RIR) for HIV positive and HIV negative women by age in the urban area of Cameroon, 2004.

**HIV positive women**				**HIV negative women**			
Age bracket (years)	Number of births **(A1)**	Woman-years at risk **(B1)**	Fertility rate (95% CI) C1 = (A1/B1)*1000	Number of births **(A2)**	Woman-years at risk **(B2)**	Fertility rate (95% CI) C2 = (A2/B2)*1000	RIR (95% CI) **C1/C2**

15–19	14	84.4	165.8 (98.1–257.9)	196	2139.9	91.6 (79.4–105.1)	1.81 (1.59–2.08)
20–24	20	177.0	113.0 (71.0–171.4)	240	1491.0	161.0 (141.6–182.3)	0.70 (0.63–0.77)
25–29	25	185.3	134.9 (89.5–196.0)	219	1151.6	190.2 (166.2–216.6)	0.71 (0.64–0.77)
30–34	6	104.2	57.6 (23.3–119.8)	140	1050.4	133.3 (113.8–155.4)	0.44 (0.35–0.52)
≥ 35	5	130.6	38.3 (14.2–83.2)	83	1470.8	56.4 (45.3–69.7)	0.68 (0.55–0.79)

**Total**	70	681.5	102.7 (80.7–129.0)	878	7079.7	124.0 (116.0–1312.4)	0.83 (0.76–0.89)

CI = confidence interval; RIR = relative inclusion ratio; HIV = Human Immunodeficiency Virus

Overall, the fertility rate in all HIV positive women (rural and urban combined) was 118.7 births per 1000 woman-years (95% CI 98.4 to 142.0) compared to 171.3 births per 1000 woman-years (95% CI 164.5 to 178.2) for HIV negative women. Consequently, the summary RIR was 0.69 (95% CI 0.62 to 0.75). These values of fertility rates are self-weighted in terms of age and rural-urban differences because the sample represents all women of reproductive age in Cameroon.

## Discussion

This study compared fertility rates in HIV-infected and uninfected women of reproductive age using population-based data. Our findings indicate that fertility rates are lower in HIV infected women compared to uninfected women in Cameroon.

Our finding of low fertility in HIV positive women is consistent with reports from other authors [3,4]. We found low fertility rates in HIV positive women of all age groups but for urban HIV-infected teenagers (that is, 15–19 year olds) who had a higher fertility rate than their corresponding HIV negative counterparts in cities. We could not find any suitable explanation to this finding. However, since the teenagers in rural areas were similar to other adult women in terms of their RIR, the cause is unlikely to be biological. We therefore attributed the high fertility in urban HIV-infected teenagers to differences in sexual and reproductive attitudes and practices. Monitoring of HIV epidemic via antenatal sentinel surveillance requires regular adjustments for many factors such as the differences in distribution of HIV across different age groups and different sub-populations, and differences in fertility rates between the infected and the uninfected women [26]. Adjustment for fertility rates is an important factor because modest changes in fertility can have profound effects on the validity of estimates from pregnant women [27]. Nicoll et al suggested that adjusting for the differences in fertility rates was sufficient in countries with overwhelming epidemics where the infection was transmitted through one source (heterosexual route) and many people are unaware of their HIV status [2]. The authors argued that it might be possible to use summary RIR in wider geographical area such as Sub-Saharan Africa. Our study reports the summary RIR for Cameroon. Since the study population was a representative sample of the all Cameroon women of reproductive age, our RIR is self-weighted for regional and age differences. Eighty three percent (83%) of Cameroonian women attend at least one antenatal visit during pregnancy [22]. However, the differences in utilization of antenatal services by HIV-positive and HIV-negative women could introduce bias in the ANC-based prevalence estimates.

Our study used fertility rates (number of births per 1000 woman years of exposure over 36 months) instead of live birth rates (number of live births per 100 woman years of exposure) as reported by Nicoll et al [2], because we consider that all pregnant women are equally likely to be tested for HIV infection irrespective of the birth outcome (dead or live births). Desgrées du Loû et al used parity as a proxy for fertility rate [28]. Parity is a cumulative measure of fertility and is insensitive to recent changes in fertility. In Zambia, ANC-based HIV estimates were found to substantially underestimate declines in HIV prevalence in the general population [29]. This was partly explained by changes in fertility-related behaviours among young women.

We found a summary RIR of 0.69 which is lower than that reported by other authors [4,28,30]. Nicoll et al reported a summary RIR of 1.03 for London and 0.80 for women else where in England and Wales [2]. The differences may be explained by socio-cultural differences of the two populations. Desgrées du Loû et al in Ivory Coast reported a summary RIR of 0.84 which is higher than our summary RIR [28]. This may be explained by the fact they used parity instead of fertility rates and collected data from antenatal clinics which may not accurately represent the whole population. Gray et al [4] is a population-based study in Uganda reported an unadjusted RIR of 0.78. Similarly, in another population-based study Terceira et al reported an unadjusted RIR of 0.78 in rural Zimbabwe [30]. The RIRs of these two studies are closer to that reported in our study and this could be explained by the fact that they all used population-based data for analysis.

The application of HIV seroprevalence from pregnant women to the whole female population needs adjustment for the differences in fertility rates among HIV-infected and HIV-uninfected women. Following earlier publications, fertility rate is now taken into consideration by the Joint United Nations Programme on HIV/AIDS (UNAIDS) when estimating and projecting HIV prevalences [31].

## Conclusion

In conclusion, fertility rates are lower in HIV infected women compared to uninfected sexually active women in Cameroon. The findings of this study support the use of summary RIR for the adjustment of HIV prevalence (among adult female population) obtained from sentinel surveillance in antenatal clinics. Continuous monitoring of the fertility rates in HIV positive and HIV negative women should be an adjunct to HIV serosurveillance because fertility rates are not static, but change over time.

## Conflict of interests

The authors declare that they have no competing interests.

## Authors' contributions

EJ Kongnyuy conceived the study, and analyzed the data, and had primary responsibility for writing this paper. CS Wiysonge assisted in the critical revision of the manuscript for important intellectual content.

## Pre-publication history

The pre-publication history for this paper can be accessed here:


